# Cell-based therapies reverse the heart failure-altered right ventricular proteome

**DOI:** 10.21203/rs.3.rs-4752035/v1

**Published:** 2024-09-06

**Authors:** Nour Makkaoui, Vidhya Prasad, Pritha Bagchi, Tiffany Carmona, Ke Li, Olivia Latham, Yuanyuan Zhang, Jingyun Lee, Cristina Furdui, Joshua Maxwell

**Affiliations:** Emory University School of Medicine; Emory University School of Medicine; Emory University School of Medicine; Emory University Emory College of Arts and Sciences; Emory University Emory College of Arts and Sciences; Wake Forest Institute for Regenerative Medicine; Wake Forest Institute for Regenerative Medicine; Wake Forest University School of Medicine; Wake Forest University School of Medicine; Wake Forest Institute for Regenerative Medicine

**Keywords:** proteomics, stem cells, heart failure

## Abstract

**Background:**

Congenital heart defects can lead to right ventricular (RV) pressure-overload and heart failure. Cell-based therapies, including mesenchymal stromal cells (MSCs) and c-kit positive cells (CPCs) have been studied clinically as options to restore heart function in disease states. Many studies have indicated these cells act through paracrine mechanisms to prevent apoptosis, promote cellular function, and regulate gene/protein expression. We aimed to determine the proteomic response of diseased hearts to cell therapy

**Methods:**

We utilized an animal model of RV pressure overload created by banding the pulmonary artery (PAB). Two weeks post-banding, bone marrow-derived mesenchymal stromal cells (MSCs) and 3 populations of CPCs (nCPCs, cCPCs, ES-CPCs) were delivered to the RV free wall. RV function and cellular retention were measured for four weeks post-injection, at which point hearts were extracted and the RV was excised for liquid chromatography and tandem mass spectrometry. Resulting RV proteomes were compared and analyzed using systems biology and bioinformatics.

**Results:**

Proteomic profiling identified 1156 total proteins from the RV, of which 5.97% were significantly changed after PAB. This disease-altered proteome was responsive to cellular therapy, with 72% of the PAB-altered proteome being fully or partially reversed by MSC therapy. This was followed by nCPCs (54%), ES-CPCs (52%), and cCPCs (39%). Systems biology and bioinformatics analysis showed MSC, nCPC, or ES-CPC cell therapy is associated with a decrease in predicted adverse cardiac effects. We also observed an effect of cell therapy on the non-altered RV proteome, however, this was associated with minor predicted pathological endpoints.

**Conclusions:**

Our data indicate MSCs, ES-CPCs, and nCPCs significantly reverse the PAB-altered proteome towards a pre-disease state. These results indicate cell-based therapies show promise in improving RV function after pressure overload through partial restoration of the disease-altered cardiac proteome.

## BACKGROUND

Nearly 1% of babies born in the US will be diagnosed with a congenital heart defect (CHD) (1). In the pediatric population, CHDs are the leading cause of right-ventricular (RV) hypertrophy and heart failure, especially in patients with defects like pulmonary stenosis, Hypoplastic Left Heart Syndrome and Tetralogy of Fallot (2). In these complex CHDs, the RV is subject to pressure overload leading to hypertrophy and, when the heart is under sustained stress, the hypertrophic response can evolve into decompensated heart failure.

Therapeutic options for RV failure are limited and much of the basic and clinical research has historically focused on the LV. Current therapeutics for RV failure including pharmacological agents, further surgical or mechanical interventions, and eventually heart transplantation at the end-stage of HF make the clinical management of patients with RV failure both costly and challenging (3, 4). The recent emergence of stem cell-based myocardial repair for the treatment of HF has provided an alternative approach. Many cell types have been investigated for their therapeutic potential in heart failure and after myocardial infarction, including but not limited to bone marrow-derived mesenchymal stromal cells (MSCs) (5, 6), cardiac-derived c-kit positive cells (CPCs) (7–10), bone marrow mononuclear cells (BMMNCs) (11, 12), and umbilical cord blood (UBC)-derived cells (13–15). These studies and others have contributed to the growing evidence supporting the therapeutic potential of cell-based therapies in children with CHD. Furthermore, recently reported clinical trials, such as ELPIS, TICAP, and PERSEUS, have illustrated long-term functional benefits in pediatric patients receiving MSCs and CPCs (5, 8, 16, 17), and another pediatric trial using autologous CPCs is currently enrolling participants (18). With the increasing number of cell-based clinical trials in CHD patients, investigations into the mechanisms of these effects are critically needed.

Recent studies have suggested that the mechanism of stem cell-mediated repair of the heart is through an indirect paracrine effect such as the release of reparative factors (19–23). This evidence suggests that cell-based repair takes place through the release of paracrine factors into the surrounding tissue that induces a number of restorative processes including myocardial protection, neovascularization, cardiac remodeling, and differentiation (24). Included in these paracrine mediators released by cell therapies are growth factors, exosomes, micro RNAs, and extracellular matrix proteins. How exactly these myriad factors may affect the native cells of the heart is an area of intense research.

In this study, we aimed to determine the response of diseased hearts to cell therapy. Using an animal model of right ventricular heart failure, we injected MSCs and 3 populations of CPCs into the heart after disease initiation. Cardiac function was assessed for 4 weeks post-injection, when hearts were excised and processed for mass spectrometry. We compared the proteomes of the RV between control hearts, diseased hearts, and hearts receiving cell therapy. We then used systems biology and bioinformatics approaches to delineate the effects and outcomes of cell therapy on the diseased hearts.

## METHODS

Because of the fact that this study involves data collected under Institutional Review Board approval, the data that support the findings of this study are available from the corresponding author on reasonable request at jtmaxwel@wakehealth.edu. This work has been reported in line with the ARRIVE guidelines 2.0.

### Human Sample Acquisition and Isolation of Human CPCs

This study was approved by the Institutional Review Board at Children’s Healthcare of Atlanta and Emory University. Human c-kit positive cells (CPCs) used in this study were isolated from right atrial appendage tissue routinely removed during surgical repair of congenital heart defects as previously described (25). Atrial tissue was transported to the laboratory in Krebs-Ringer solution containing 35 mM NaCl, 4.75 mM KCl, 1.2 mM KH2PO4, 16 mM Na2HPO4, 134 mM sucrose, 25 mM NaHCO3, 10 mM glucose, 10 mM HEPES, and 30 mM 2,3-butanedione monoxime, pH = 7.4 with NaOH. Child CPCs (cCPCs) are categorized as being isolated from patients aged 12 months to 5 years. Neonatal CPCs (nCPCs) are categorized as being isolated from patients aged 1 day to 1 month (25). Both populations of CPCs were created by pooling isolated cells from 3 distinct donors to prevent patient-specific effects. Human bone marrow-derived mesenchymal stromal cell (MSCs) were purchased from Lonza (PT-2501, Morrisville, NC). Two distinct batches of cells (19TL155677, 18TL113327) were pooled together to prevent batch or patient-specific effects.

### Cell Culture

MSCs were maintained in culture in Mesenchymal Stem Cell Basal Medium (PT-3238, Lonza, Morrisville, NC) supplemented with Mesenchymal Stem Cell Growth Medium SingleQuots (PT-4105, Lonza, Morrisville, NC) containing Mesenchymal Stem Cell Growth Supplement, L-glutamine, and GA-1000. All populations of CPCs were maintained in culture in Ham’s F12 medium (11765054, ThermoFisher Scientific, Waltham, MA) supplemented with 10% of fetal bovine serum (FBS), 100 U/ml of penicillin/streptomycin, 2 mmol/l of l-glutamine, and 0.01 μg/ml of basic fibroblast growth factor (bFGF). cCPCs also underwent a protocol of electrical stimulation (ES-CPCs) as previously reported (26). For electrical stimulation of cCPCs, cells were seeded onto 6-well dishes at ~1,000,000 cells per well in calcium-supplemented media (CPC culture media plus 2 mmol/l CaCl2). A C-dish electrode array in conjunction with C-Pace Electrical Stimulation System (Ion Optix, Westwood, MA) was used to apply chronic electrical pulses to the cells at 1 Hz frequency, 10 ms duration, and 10 V amplitude for 7 days. Media was replaced every 24–48 hours.

### Rat Pulmonary Artery Banding (PAB) Model

All animal experiments were performed with the approval of the Institutional Animal Care and Use Committee of Emory University and conform to the guidelines from the NIH Guide for the Care and Use of Laboratory Animals. Male adolescent (3–4 weeks old) athymic rats (Crl:NIH-Foxn1 rnu) (~150 g) were obtained from Charles River Laboratories (Wilmington, MA). All rats exhibited normal RV function on echocardiography at the time of pulmonary artery banding (PAB) surgery (3–4 weeks old). Rats were anesthetized with 2% isoflurane until no response from toe pinch reflex and a limited left thoracotomy was performed to expose the pulmonary artery (PA). The PA was dissected from the aorta and partially ligated over an 18-gauge angiocatheter. The sizer was then promptly removed to allow for antegrade flow through the banded area, and thoracotomy performed was closed under positive pressure ventilation to evacuate pleural air (n = 20). Sham operated control (CTL) animals underwent the same procedure without banding the pulmonary artery (n = 4). Animals were double housed.

### Echocardiography

Transthoracic echocardiography was performed prior to surgery and at 2 weeks, 4 weeks, and 6 weeks post-banding using a Vevo 2100 digital high-frequency ultrasound system (FujiFilm Visualsonics, Toronto, ON, Canada) equipped with a probe (MS250) suited for rat imaging. Tricuspid annular plane systolic excursion (TAPSE) was measured in the apical four-chamber view in M-mode. As previously shown, this surgery produces severe right ventricular dysfunction within two weeks post-banding (26, 27). Right ventricular dysfunction was confirmed as at least a 35% reduction in TAPSE. Ultrasound acquisition and analysis was performed blinded with only PI of study knowing group allocation.

### Cell Therapy Treatments

Two weeks post-banding, animals were randomized into cohorts without (Sham and PAB) or with cell therapy (MSC, cCPC, nCPC, ES-CPC). All cells for therapy were expanded to passage 4 in culture to normalize passage number across treatment groups. Cells were harvested in sterile saline and labeled with DiR (1,1’-dioctadecyl-3,3,3,’,3’-tetramethylindotricarbocyanine iodide; Thermo Fisher, Waltham, MA) per manufacturer’s protocol. Cells were injected under echocardiographic guidance into the RV free wall using a 27-gauge BD Insulin Syringe with 12.7 mm BD Micro-Fine short bevel needle mounted on a stereotactic frame (BD Medical Technology) into 3 spots on the right ventricular free wall, totaling 500,000 cells per heart and ~50 μL per injection. Successful delivery of CPCs into the rat myocardium was confirmed by echocardiography. The PAB cohort received saline injections equal to the volume of cell injections for the cell therapy groups. Cell retention was tracked using an IVIS Spectrum in vivo imaging system (Perkin Elmer, Waltham, MA). DiR fluorescence in the rat heart was measured as radiant efficiency and compared between rats as percentage retention (100% on day 0) over time (26, 27). Animal cohorts are identified as CTL (Sham), PAB (banded, saline injected), MSC (PAB + MSC injection), nCPC (PAB + nCPC injection), cCPC (PAB + cCPC injection), or ES-CPC (PAB + ES-CPC injection). N=4 animals/hearts used for each group except MSC (n = 3). No inclusion or exclusion criteria were used for animals or resulting data points. All animals were processed simultaneously for treatments and analysis.

### Mass Spectrometry

Rats were anesthetized with ketamine (0.1 mg/g) and xylazine (0.01 mg/g), and hearts were excised for analysis at 6 weeks post-banding or sham surgery. Hearts were rinsed and flushed with cold phosphate buffered saline, and the right ventricle was excised and snap frozen in liquid nitrogen. For the protein isolation, 300 μL of urea lysis buffer (8 M urea, 10 mM Tris, 100 mM NaH_2_PO_4_, pH 8.5), including 3 μL (100x stock) HALT(-EDTA) protease and phosphatase inhibitor cocktail (Pierce) was added to the tissue. Samples were sonicated (Sonic Dismembrator, Fisher Scientific) 3 times for 5 sec each with 5 sec intervals of rest at 30% amplitude to disrupt nucleic acids and were subsequently centrifuged at 4° C. Protein concentration was determined by the bicinchoninic acid (BCA) method, and samples were frozen in aliquots at −80°C. Protein homogenates (100 μg) were treated with 1 mM dithiothreitol (DTT) at room temperature for 30 min, followed by 5 mM iodoacetimide at room temperature for 30 min in the dark. Protein samples were digested with 1:100 (w/w) lysyl endopeptidase (Wako) at room temperature for overnight. Next day, samples were diluted with 50 mM NH_4_HCO_3_ to a final concentration of less than 2 M urea and were further digested overnight with 1:50 (w/w) trypsin (Promega) at room temperature. Resulting peptides were desalted with HLB column (Waters) and were dried under vacuum.

The data acquisition by LC-MS/MS protocol was adapted from a published procedure (Seyfried, Dammer et al. 2017) and was performed by the Integrated Proteomics Core Facility at Emory University. Derived peptides were resuspended in 100 μL loading buffer (0.1% trifluoroacetic acid). Peptide mixtures (2 uL) were separated on a self-packed C18 (1.9 μm, Dr. Maisch, Germany) fused silica column (15 cm × 100 μm internal diameter (ID); New Objective, Woburn, MA) attached to an EASY-nLC^™^ 1200 system and were monitored on a Q-Exactive Plus Mass Spectrometer (ThermoFisher Scientific, San Jose, CA). Elution was performed over a 56 min gradient at a rate of 700 nL/min (buffer A: 0.1% formic acid in water, buffer B: 0.1% formic acid in acetonitrile): The gradient started with 1% buffer B and went to 40% in 56 minutes, then increased from 40–99% within 1 minute and finally staying at 99% for 3 minutes. The mass spectrometer cycle was programmed to collect one full MS scan followed by 20 data dependent MS/MS scans. The MS scans (400–1600 m/z range, 1×10^6^ AGC target, 100 ms maximum ion time) were collected at a resolution of 70,000 at m/z 200 in profile mode. The HCD MS/MS spectra (2 m/z isolation width, 28% collision energy, 1×10^5^ AGC target, 50 ms maximum ion time) were acquired at a resolution of 17,500 at m/z 200. Dynamic exclusion was set to exclude previously sequenced precursor ions for 20 seconds within a 10 ppm window. Precursor ions with + 1, and + 7, or higher charge states were excluded from sequencing.

Label-free quantification analysis was adapted from a published procedure (28). Spectra were searched using the search engine Andromeda, integrated into MaxQuant, against rat Uniprot/Swiss-Prot database (8097 target sequences). Methionine oxidation (+ 15.9949 Da), asparagine and glutamine deamidation (+ 0.9840 Da) and protein N-terminal acetylation (+ 42.0106 Da) were variable modifications (up to five allowed per peptide); cysteine was assigned as fixed carbamidomethyl modification (+ 57.0215 Da). Only fully tryptic peptides with up to two miscleavages were considered in the database search. A precursor mass tolerance of ± 20 ppm was applied before mass accuracy calibration and ± 4.5 ppm after internal MaxQuant calibration. Other search settings included a maximum peptide mass of 6,000 Da, a minimum peptide length of six residues and 0.05-Da tolerance for high resolution MS/MS scans. The FDR for peptide spectral matches, proteins and site decoy fraction was set to 1%. Quantification settings were as follows: match full MS1 peaks between runs; use a 0.7-min retention time match window after an alignment function was found with a 20-min retention time search space. The LFQ algorithm in MaxQuant was used for protein quantitation. The quantitation method considered only razor and unique peptides for protein level quantitation. Data was prepared for presentation using Perseus software, including heat maps and principal component analysis. Proteins were consider significantly upregulated or downregulated at ± 1.3-fold, *p* < 0.05. The mass spectrometry proteomics data have been deposited to the ProteomeXchange Consortium via the PRIDE partner repository with the dataset identifier PXD051582 (Reviewer account details: Username: reviewer_pxd051582@ebi.ac.uk, Password: ewve677t.)

### Proteomic Ingenuity Pathway Analysis and Gene Ontology

Differentially expressed proteins were submitted to Ingenuity Pathway Analysis (IPA, QIAGEN Bioinformatics, Hilden, Germany) for pathway and network analysis. Disease and cell therapy-associated networks (Networks tab), upstream regulators or proteome changes (Upstream Analysis tab, Upstream Regulators sub-heading), and enriched functional annotations (Diseases & Functions tab) associated with ‘Cardiovascular System Development and Function’ and ‘Cardiovascular Disease’ (Diseases and Bio Functions sub-heading) and cardiac adverse effects (‘Cardiotoxicity’ categories under the Tox Functions sub-heading) were derived, using Fisher’s exact test for p-value calculations. DAVID Functional Annotation Clustering Tool (http://david.abcc.ncifcrf.gov/) with Benjamini-Hochberg FDR correction for p-value calculations was used for gene ontology analysis.

### Statistical Analysis

Data are presented as mean ± SD unless otherwise noted in the legend. Statistical analysis was performed using unpaired t-test and ANOVA (GraphPad Prism, v9.2.0) as noted. Differences were considered statistically significant at P < 0.05 or as noted in the figure legend.

## RESULTS

### Right Ventricular Proteome Resolution and Responsiveness to Cell Therapy

**Figure 1:**
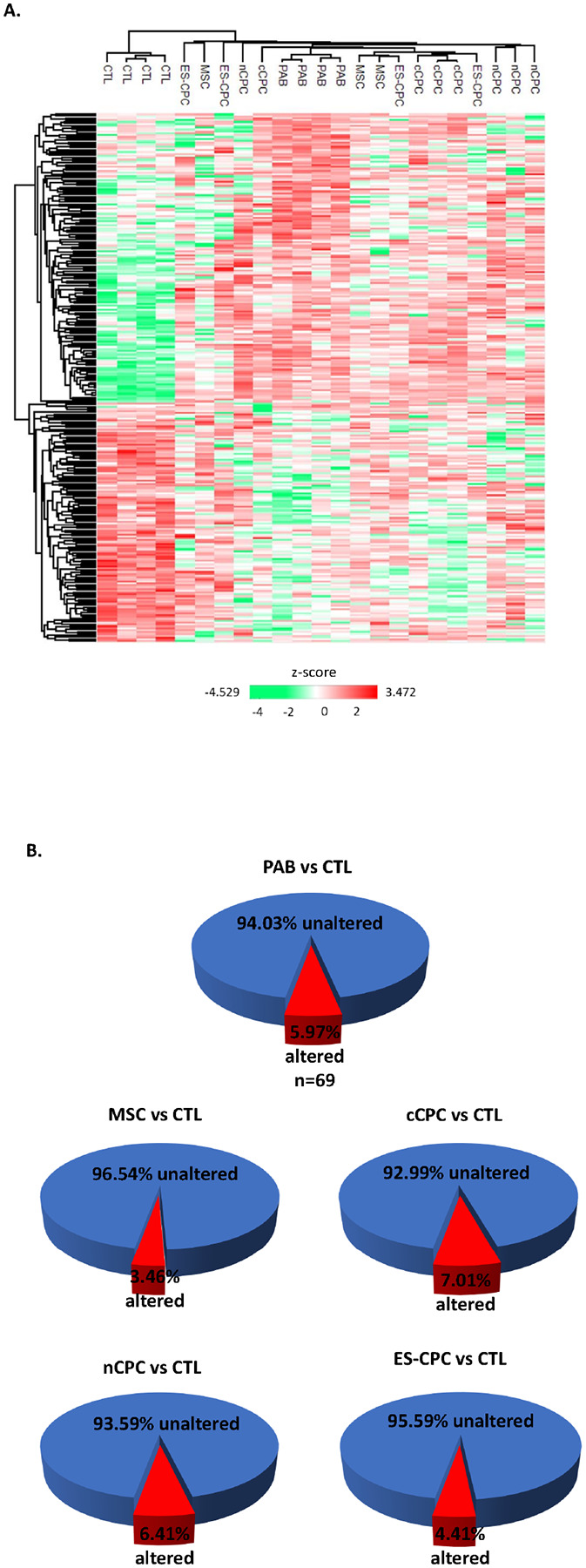
Cell therapy can affect the disease-altered cardiac proteome. **A**. Heatmap of protein expression z-scores of the 1156 identified right ventricular proteins in CTL, PAB, MSC, nCPC, cCPC, and ES-CPCs groups (n=3–4). **B**. Pie charts of significant changes in cardiac proteome in disease and after cell therapy. Of the 1156 proteins identified, 5.97% (n=69) were altered significantly between CTL and PAB (24 downregulated and 45 upregulated). Pie charts for the percentage of significantly altered proteins in cell therapy groups are also shown vs. CTL for MSCs (3.46%; 12 down, 28 up), cCPCs (7.01%; 27 down, 54 up), nCPCs (6.41%; 28 down, 46 up), and ES-CPCs (4.41%; 18 down, 33 up). Proteins were considered significantly changed at ±1.3 fold, p<0.05. n=4 (CTL, PAB, nCPC, cCPC, ES-CPC), n=3 (MSC).

**Figure 2:**
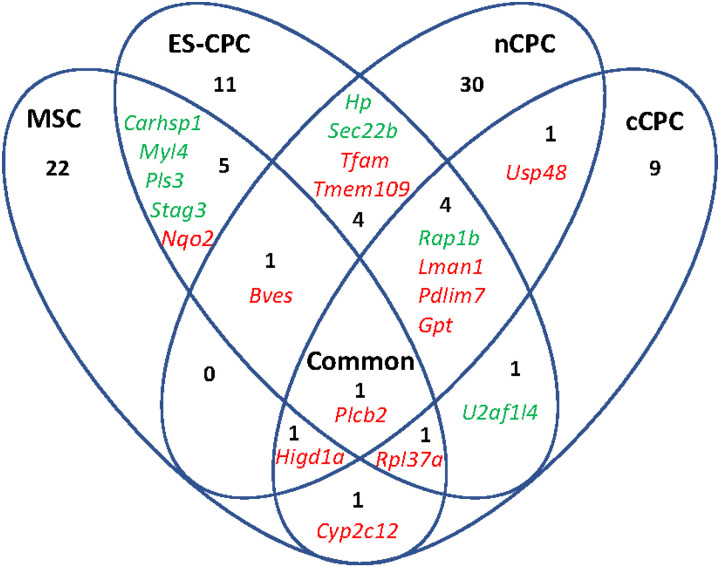
Common and unique proteins altered by cell therapy. Venn diagram of proteins altered by cell therapy by comparing cell therapy proteomes to PAB proteome. Red indicates upregulated proteins, green indicated downregulated proteins. The numbers in each intersection represent the number of proteins in this intersection. All cell therapy groups shared 1 common protein (PLCB2).

To test whether cell therapy affects the disease altered cardiac proteome, we first created an animal model of pediatric right ventricular (RV) heart failure due to pressure overload by pulmonary artery banding (PAB). We and others have shown PAB rapidly induces right ventricular hypertrophy and failure of the RV in rats, with RV functional parameters significantly reduced 2 weeks post-banding (23, 25–27, 29). We then applied cell therapy in the form of four populations of cells (MSCs, nCPCs, cCPCs, and ES-CPCs) into the RV free wall of juvenile rats that had undergone PAB surgery at 2 weeks post-banding. Animal cohorts and proteomes are identified as CTL (Sham), PAB (banded, saline injected), MSC (PAB + MSC injection), nCPC (PAB + nCPC injection), cCPC (PAB + cCPC injection), or ES-CPC (PAB + ES-CPC injection). RV protein isolation and identification by label-free tandem mass spectrometry identified 1156 proteins. Of these 1156 proteins, PAB significantly altered 69 proteins (5.97%), with 24 downregulated and 45 upregulated. Among these significantly changed proteins were common markers of cardiac hypertrophy and heart failure including upregulation of atrial natriuretic peptide (NPPA) (30), skeletal muscle alpha-actin (ACTA1) (31), four-and-a-half LIM domain protein 1 (FHL1) (31), and myosin heavy chain 7 (MYH7) (32), and downregulation of four-and-a-half LIM domain protein 2 (FHL2) (33) (**Additional file 1: Supplemental Table 1 and Additional file 3**). Comparison of the PAB + cell therapy proteomes to the CTL proteome revealed that MSCs (3.46%; 12 down, 28 up) and ES-CPCs (4.41%; 18 down, 33 up) reduced the proteome alterations, while nCPCs (6.41%; 28 down, 46 up) and cCPCs (7.01%; 27 down, 54 up) increased the proteome alterations (Fig. 1A – 1B, **Additional file 1: Supplemental Table 2 and Additional file 3**). We next compared the cell therapy proteomes to the PAB proteome to determine which proteins were significantly changed by cell therapy (**Additional file 1: Supplemental Table 2 and Additional file 3**). Figure 2 shows the proteins significantly changed by cell therapy specific to each cell therapy and also shared between cell therapies. MSCs and ES-CPCs shared the highest number of proteins in common with 5, followed by ES-CPCs and nCPCs which shared 4 common proteins, while all three CPC groups (nCPC, cCPC, and ES-CPC) also shared 4 proteins in common. Interestingly, all four cell therapy groups shared the upregulation of PLCB2 protein. Gene ontology (GO) enrichment analysis was performed on the significantly changed proteins in PAB vs CTL groups and also between cell therapy and PAB groups to gain insights into the cellular functions and biological processes that are affected in PAB and after cell therapy (**Additional file 2: Supplemental Fig. 1 and Additional file 3**). We found that upregulated proteins in PAB were significantly enriched with GO categories linking to heart/muscle contraction, sarcomere organization, cardiac hypertrophy, and response to hypoxia, whereas downregulated proteins in PAB were overrepresented with GO terms associated with responses to mechanical stimulus, fatty acid beta-oxidation, and cytoskeleton organization. Cell therapy and PAB group comparisons showed significantly changed proteins in the cell therapy groups were significantly enriched with GO categories including development and regeneration processes and the response to ischemia.

### Cell Therapy Effect on the Reversal of the Diseased RV proteome

**Figure 3:**
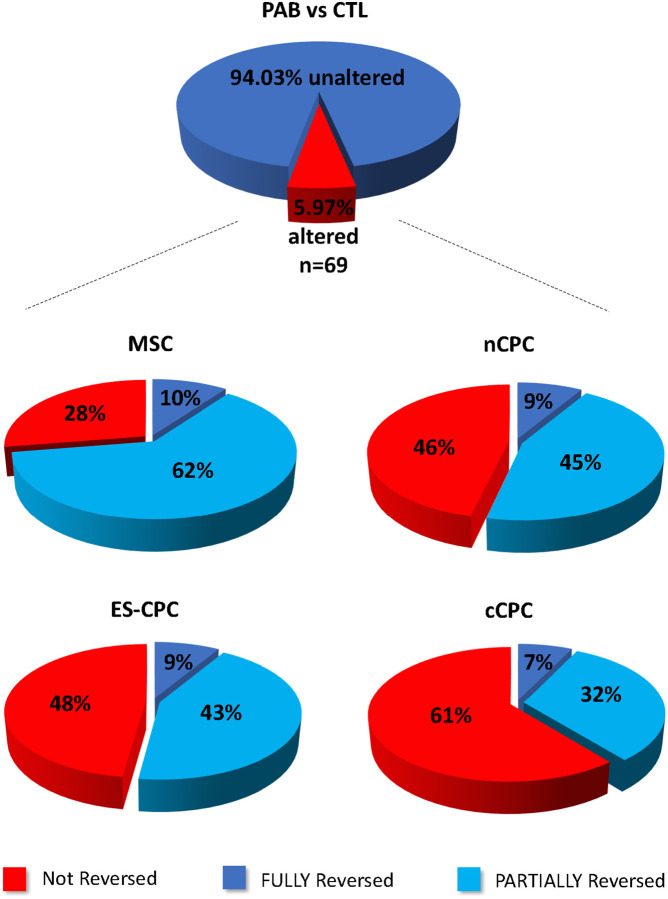
Cell therapy reversed a majority of the proteins altered by PAB. The 69 proteins significantly altered by PAB (±1.3 fold, p<0.05) were further examined by expression level fold change in the cell therapy groups. These 69 proteins were determined to be either not reversed by cell therapy (red), fully reversed by cell therapy (dark blue), or partially reversed by cell therapy (light blue).

**Figure 4:**
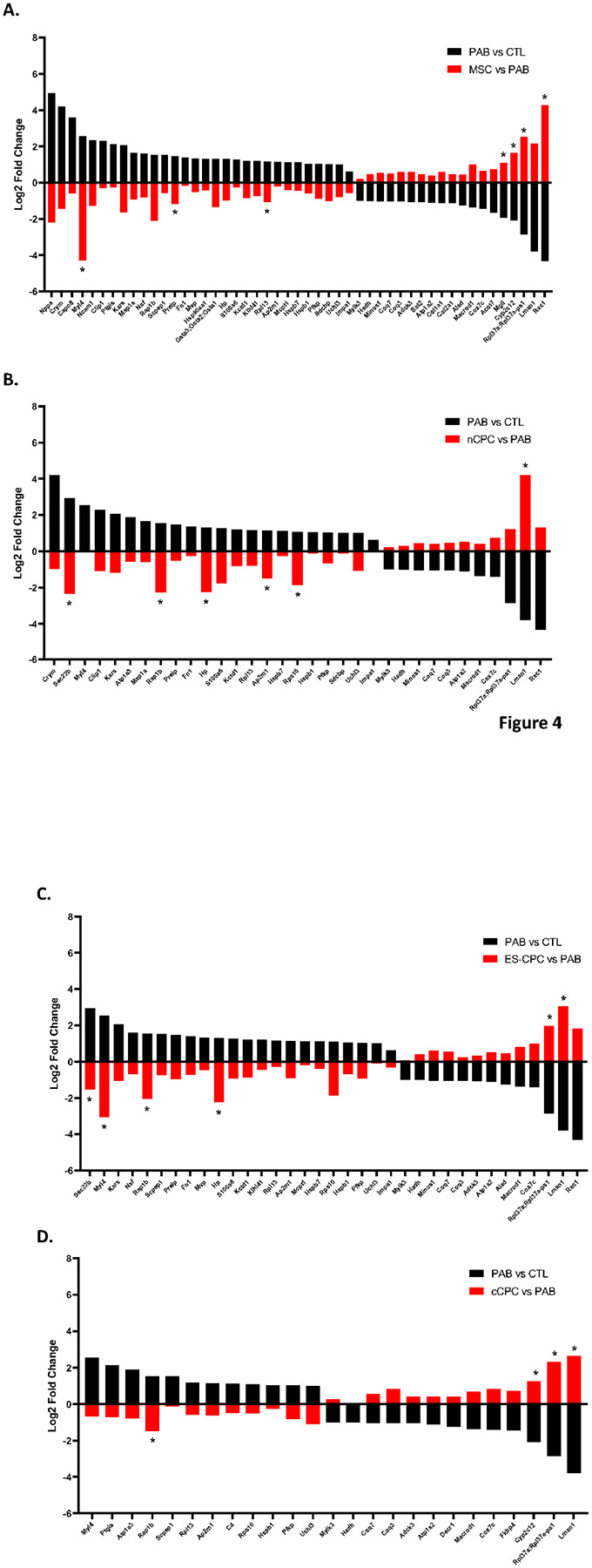
Bar graphs of fully and partially reversed proteins for each cell therapy group. The fold change for each fully and partially reversed protein for each cell therapy group (MSC, nCPC, ES-CPC or cCPC) vs PAB (red bars) and the corresponding PAB vs CTL fold change (black bars) are plotted. Only the 69 proteins significantly changed (±1.3 fold, p<0.05) by PAB were included in the analysis. Asterisk indicates proteins fully reversed by cell therapy.

Further examination of the 69 proteins significantly altered by PAB, showed varying degrees of expression reversal by cell therapy when compared to the PAB proteome (Fig. 3). These proteins were individually examined in each cell therapy group and classified as either fully reversed (significant vs PAB and not significant vs CTL), partially reversed (not significant vs CTL but did not reach statistical significance vs. PAB, or significant vs PAB but still significant vs CTL), or not reversed (not significant vs PAB and significant vs CTL) (34). Classification of these proteins revealed that MSC therapy fully or partially reversed the highest percentage of proteins at 10% and 62%, respectively. This was followed by nCPC therapy at 9% fully and 45% partially reversed, then by ES-CPC therapy at 9% fully and 43% partially, and finally by cCPC therapy at 7% fully and 32% partially. Only cCPC therapy showed a majority of the proteins not reversed at 61% (Fig. 3). MSC therapy targeted 67% of the downregulated proteins and 83% of the upregulated proteins, while the CPC therapy populations interestingly had similar targeting numbers with all three populations targeting 58% of the downregulated proteins and nCPC therapy targeting 51%, ES-CPC targeting 49%, and cCPC targeting 21% of the upregulated proteins. The specific fold changes for each protein fully and partially reversed by cell therapy compared to PAB vs CTL fold changes are show in Fig. 4. The overall reversal (fully + partially) was 72% for MSCs, 54% for nCPCs, 52% for ES-CPCs, and 39% for cCPCs.

GO enrichment analysis was performed on the fully and partially reversed proteins in the cell therapy groups to gain insights into the cellular functions and biological processes that are affected by cell therapy (**Additional file 2: Supplemental Fig. 2 and Additional file 3**). We found that all cell therapy groups were significantly enriched with GO categories linking to cardiac muscle contraction and the force of heart contraction.

**Figure 5:**
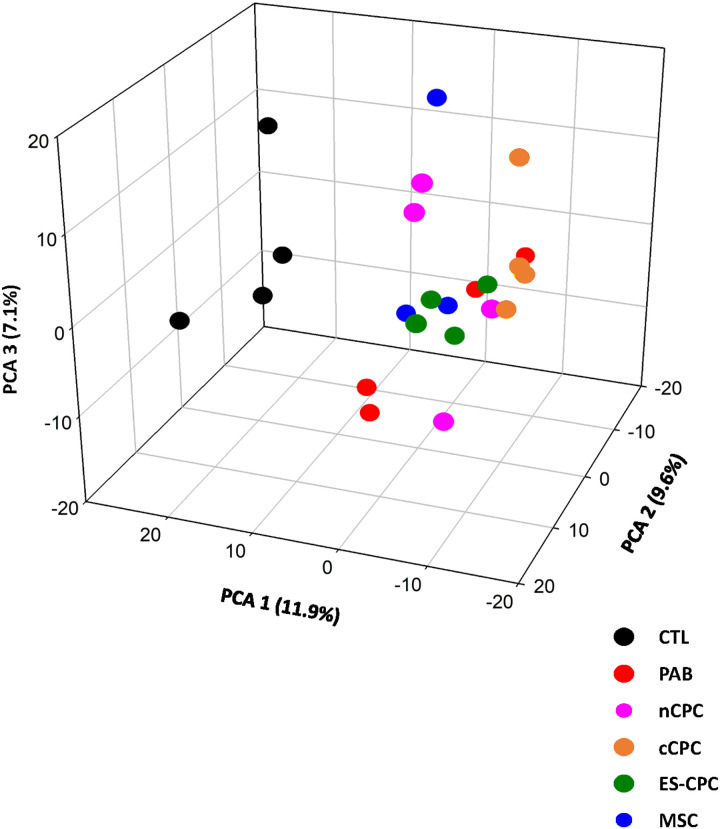
Principle Component Analysis of RV proteomes. The 3D PCA plot represents the differential protein expression of the proteomes from Sham (black, n=4), PAB (red, n=4), nCPC (purple, n=4), cCPC (orange, n=4), ES-CPC (green, n=4), and MSC (blue, n=3). PCA segregated the proteomes into distinct regions in the plot. Component 1 represents 11.9%, Component 2 represents 9.6%, and Component 3 represents 7.1% of total data.

In order to compare the overall proteomes of the 6 groups, we performed Principle Component Analysis (PCA) on the entire protein data for each sample. PCA segregated the RV proteomes into distinct clustered regions in the principle component space, with clear separation between the CTL and PAB proteomes (Fig. 5). cCPC samples clustered closely with the PAB samples, indicating these two populations were still relatively similar. As we have shown above, cCPC cell therapy reversed the fewest amount of PAB-altered proteins. In contrast, MSC samples clustered slightly towards the CTL groups, indicating a redirection of the MSC-treated proteomes towards the pre-disease (CTL) state. nCPCs and ES-CPCs shifted their respective proteomes slightly away from the diseased proteome. Taken together, these data indicate that cell therapy in the form of nCPCs, ES-CPCs, and MSCs can direct the PAB-altered RV proteome away from the disease state, albeit to varying degrees.

### The Reversed Proteome is Associated with a Decrease in Disease Associations

**Figure 6:**
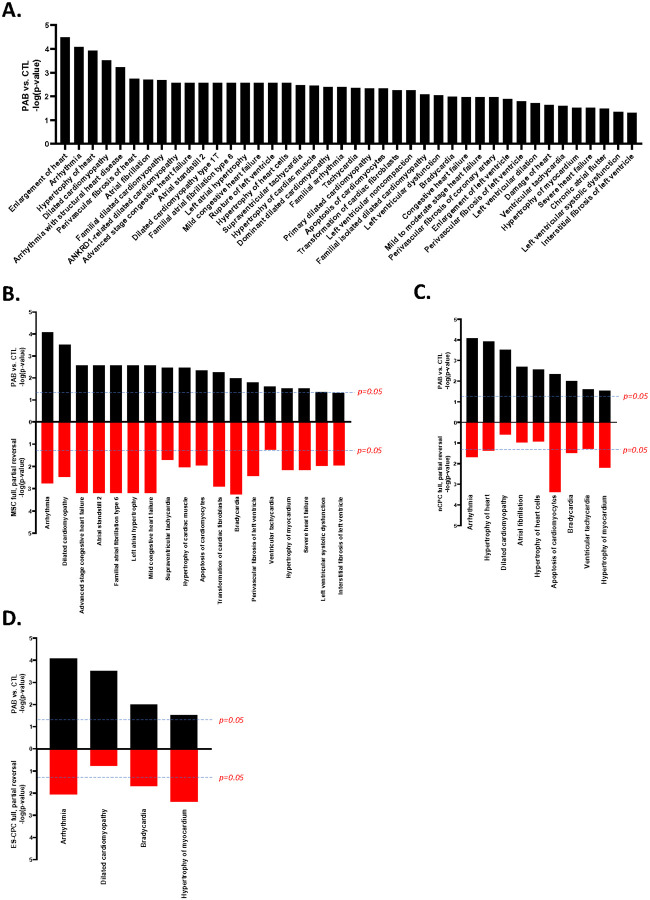
Cell therapy reduced the predicted cardiac adverse effects. (**A**) Ingenuity Pathway Analysis (IPA) identified 42 cardiac disease associations in the PAB-altered RV proteome. Further IPA of the reversed sub-proteomes from the cell therapy groups predicted reversal of many of these disease associations by MSC (**B**), nCPC (**C**), or ES-CPC (**D**) therapies. Black bars represent PAB vs CTL, red bars represent cell therapy fully and partially reversed sub-proteomes. Dashed lines in B-D indicate significance level of p=0.05.

To further investigate the importance of the total proteomes and reversed sub-proteomes on cardiac function, we performed ingenuity pathway analysis (IPA) on these proteomes from CTL, PAB, and cell therapy animals. Analysis of the PAB proteome revealed 42 predicted ‘Cardiotoxicity’ effects, prioritizing cardiac dilation and enlargement, arrhythmia, and fibrosis (Fig. 6A). Investigation of the reversed sub-proteomes from the cell therapy groups predicted that MSC, ES-CPC and nCPC therapies generally targeted cardiac dilation, hypertrophy, enlargement, and arrhythmias as part of their reversed sub-proteomes. Specifically, MSC therapy predicted a reversal of 18 adverse effects, followed by nCPC (9) and ES-CPC therapy (4) (Figs. 6B–D). cCPC therapy did not predict reversal of any cardiac-related adverse effects.

### Effect of Cell Therapy on the Non-Altered RV Proteome

**Figure 7:**
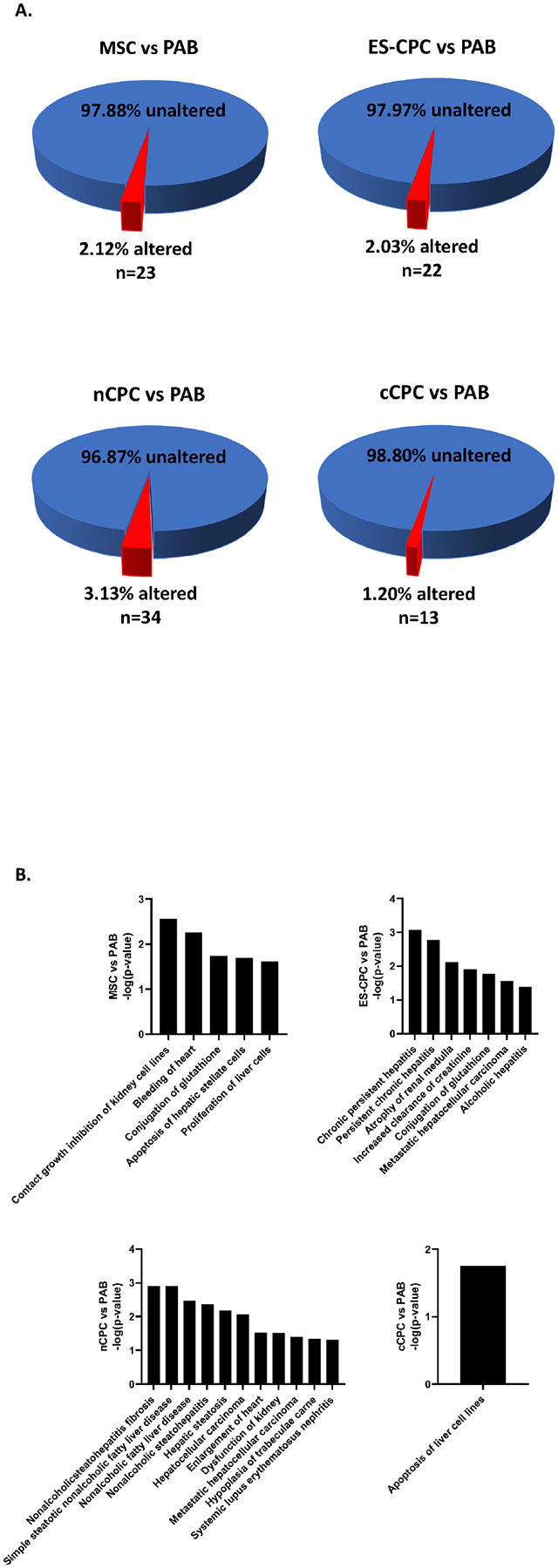
Cell therapy can affect the non-altered RV proteome. Analysis of the 1087 proteins not significantly altered by PAB revealed effects of cell therapy. (**A**) Pie charts of the percentage of these proteins that were either altered or unaltered by MSC, ES-CPC, nCPC, or cCPC cell therapy. (**B**) Summary bar graphs for each cell therapy group and the associated predicted adverse effects of proteomic changes induced by cell therapy on the non-altered RV proteome as determined using Ingenuity Pathway Analysis.

Cell therapy also had an effect on proteins not altered by disease. Of the 1087 proteins not altered by PAB (non-altered RV proteome), nCPC therapy altered 3.13% (n = 34), MSC therapy altered 2.12% (n = 23), ES-CPC therapy altered 2.03% (n = 22), and cCPC therapy altered 1.20% (n = 13) (Fig. 7A). IPA was used to determine the predicted adverse effects of cell therapy on the non-altered RV proteome, which were revealed to be relatively minimal in scope compared to the adverse effects of our disease state (Fig. 7B). Cardiac-specific adverse effects were only found in two of our cell therapy groups. MSC therapy predicts bleeding of the heart, while nCPC therapy predicts enlargement of the heart. In general, the majority of the non-altered RV proteome affected by cell therapy is predicted to be related to non-cardiac effects for all cell therapies.

### Effect of Cell Therapy on RV Function

**Figure 8:**
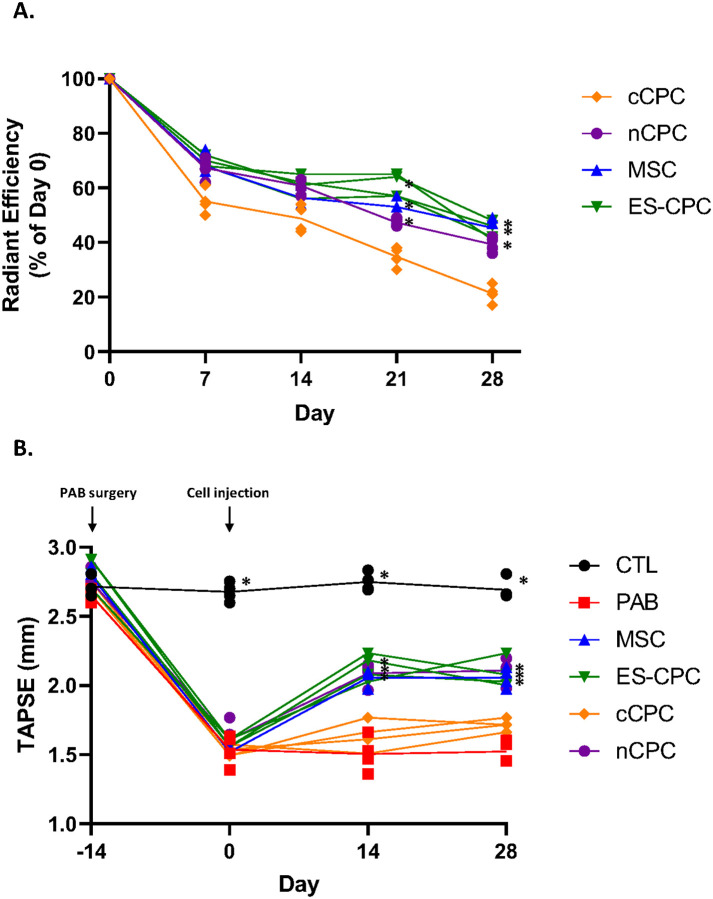
Cell therapies are retained in the heart and can affect RV function. **(A)** Summary time course of fluorescence in PAB rat hearts injected with DiR-labeled MSCs, cCPCs, nCPCs, or ES-CPCs. Values are normalized to day 0 fluorescence values and plotted as percentage. Data are presented as individual values. *, p ≤ .05 vs. cCPC. (**B**) Summary time course data of tricuspid annular plane systolic excursion (TAPSE) measurements in experimental animal cohorts. Animal cohorts are identified as CTL (Sham, n=4), PAB (banded, saline injected, n=4), MSC (PAB + MSC injection, n=3), nCPC (PAB + nCPC injection, n=4), cCPC (PAB + cCPC injection, n=4), or ES-CPC (PAB + ES-CPC injection, n=4). All animals underwent PAB surgery at Day −14 and cell therapy cell populations were injected into the RV free wall 2 weeks post-PAB (Day 0). Echocardiography was performed biweekly from Day −14. Data are presented as individual values. *, p ≤ .05 vs. PAB.

In addition to the proteomic data obtained, we also tracked RV function and cellular therapy retention over the duration of our study. Prior to injection, cells were fluorescently labeled and retention was quantified weekly after cell injection (Fig. 8A). MSCs, ES-CPCs, and nCPCs were all retained at similar levels, while cCPCs were found to be present in hearts at significantly lower levels compared to the other cell types at days 21 and 28, consistent with our previous data (25–27). We also examined the ability of cell therapy to restore RV function after PAB. Echocardiographic analysis at days 14 and 28 post-injection showed that animals receiving MSCs, ES-CPCs, or nCPCs exhibited significantly improved TAPSE compared to saline injected PAB animals, while cCPCs showed no significant improvement in TAPSE at 14 or 28 days post-injection as we have previously reported (Fig. 8B) (25–27).

## DISCUSSION

This study represents the first head-to-head comparison of multiple cell therapy populations in a translationally-relevant animal model of RV failure. We observed that the RV proteome was significantly altered by pressure-overload induced by PAB, and it retained plasticity allowing disease alteration reversal by cell therapy. The cell therapies did not specifically target the disease-altered proteome, as non-altered proteins were also affected by cell therapy. However, extensive reversal of the disease-altered proteome was observed with most of the cell therapies, along with a reduction in the predicted cardiotoxic effects revealed by IPA. Our results demonstrate that MSCs, ES-CPCs, and nCPCs significantly reverse the PAB-altered proteome towards a pre-disease state, and provide further insights into the mechanism of cell-based therapies for cardiac disease.

Of the four cell therapy populations tested, MSCs showed the highest reversal percentage of disease-altered proteins and the largest reduction in predicted adverse cardiac effects (Figs. 4 & 6). Importantly, MSCs, ES-CPCs, and nCPCs also all showed significant and similar RV function improvements at 2- and 4-weeks post-injection compared to PAB only animals (Fig. 8). However, these three cell populations also affected a relatively high percentage of the non-altered proteome compared to the cCPC therapy group (Fig. 7). Despite this, there were relatively few predicted disease associations for this non-altered RV proteome affected by MSC, nCPC, or ES-CPC therapy (Fig. 7). While the efficacy of the various cell therapies for reversing the disease-altered proteome may be a critical parameter when selecting a cell population for therapeutic implementation, the effects on the non-altered proteome for all cell types is an important consideration for future pre-clinical and clinical studies. Additionally, the changes to the non-altered proteome by cell therapies showed disease associations related to renal and hepatic function, which would become relevant if the injected cells were to either be cleared to the liver or kidneys or release factors that could translocate to these organs and affect protein expression. Future *in vivo* studies will shed light on the prevalence of any of these predicted adverse effects of cardiac cellular therapy.

Prior work has shown that the reparative potential of human CPCs is age-dependent, with neonatal CPCs (nCPCs, isolated from patients aged 1 day to 1 month) exerting the maximum beneficial effect compared with child CPCs (cCPCs, isolated from patients aged 12 months to 5 years) (25). Our results here further support those findings. We observed a higher reversal percentage of the disease-altered proteome and fewer predicted cardiac adverse effects with nCPC treatment compared to cCPC treatment. In this current study, this could be a result of the significantly reduced retention of cCPCs we observed at Days 21 and 28 post-injection compared to the other three cell types tested.

We have previously shown that *ex vivo* modification of cCPCs with electrical stimulation (producing ES-CPCs) could enhance the efficacy and retention of these cells in a PAB animal model (26). We also found that ES-CPCs released higher levels of pro-reparative factors *in vitro* compared to cCPCs. In this study, conditioning cCPCs with 7 days of electrical stimulation enhanced the ability of the resulting ES-CPCs to reverse the disease-altered proteome (Fig. 3). Our results presented here are consistent with our previous report and further show that *ex vivo* modification of cCPCs with electrical stimulation enhances the reparative effects of this cell population (26). Interestingly, ES-CPCs seem to have more in common with nCPCs, as indicated by analysis of their reversed proteins and proteome clustering by PCA (Figs. 3 & 5). These data highlight the utility of *ex vivo* modifications in improving cell-based therapy efficacy.

Recently, a similar reversal of the disease-altered cardiac proteome was shown in an elegant study using cadiopoietic stem cell-based therapy in a mouse myocardial infarction (MI) model (34). Here, cardiopoietic stem cells were able to either partially or fully reversed nearly 65% of the MI-altered cardiac proteome. Furthermore, pathway analysis showed an inverse correlation between the predicted MI- vs cell therapy-induced activation or inhibition of upstream regulators and also showed reversal of many disease functional associations and adverse cardiac effects with cell therapy. In a follow up study, the stem cell secretome was linked to many of the proteomic and functional changes observed, suggesting therapeutic efficacy of cell-based therapies is inherent to the cell secretome (35). While not directly tested in this study, we have previously linked the stem cell secretome to cell-based therapy mechanism and efficacy (22, 23, 26, 27). We hypothesize that soluble factors released by cell therapies can act on native cardiac cells to induce changes in gene and protein expression, and this could be the mechanism by which the cell-based therapies tested here are able to reverse the disease-altered proteome. Our results presented here further support that mechanism and point toward the importance of paracrine-based regenerative effects of human CPCs and MSCs. Future studies into identifying the bioactive factors in the cell therapy secretome and how these factors may cause transient and sustained effects on the structure and function of native cardiac cells are warranted. Additionally, investigations into various dosing regimens and combination cell therapies may provide superior reversal of the disease altered proteome from what we have observed here.

### Limitations

The limitations of this study include the testing of only one dosage (500,000 cells, one injection) and one endpoint (4 weeks post-injection). Additionally, the depth of our proteomic data, although sufficient, could have been enhanced by using more sophisticated tandem mass spectrometry equipment for identification and quantification of more protein species. We also did not gather any data on the spatial profile of the protein expression changes and what specific cell types of the heart (i.e. cardiac myocytes, cardiac fibroblasts, cardiac endothelial cell, etc.) are contributing to the global changes in protein expression we observed. Finally, we did not identify the molecular mechanism (i.e. miRs, growth factors, etc) of how cell therapy is able to reverse the disease-altered proteome. We envision related future studies will be aimed at determining the dosage-related changes in the cardiac proteome, if multiple cell injections enhance our observed effects, and what happens to the cardiac proteomes at > 4 weeks post-injection of cells.

## CONCLUSIONS

In this study, we used proteomic profiling to identify proteins altered by right ventricular pressure overload with and without cellular therapy. The disease-altered proteome was responsive to cellular therapy, with 72% of the PAB-altered proteome being fully or partially reversed by MSC therapy. This was followed by nCPCs (54%), ES-CPCs (52%), and cCPCs (39%). Systems biology and bioinformatics analysis showed MSC, nCPC, or ES-CPC cell therapy is associated with a decrease in predicted adverse cardiac effects. We also observed an effect of cell therapy on the non-altered RV proteome, however, this was associated with minor predicted pathological endpoints. Our data indicate MSCs, ES-CPCs, and nCPCs significantly reverse the PAB-altered proteome towards a pre-disease state and indicate that cell-based therapies show promise in improving RV function after pressure overload through partial restoration of the disease-altered cardiac proteome.

## Data Availability

All reported data have been obtained from experiments performed in the author’s laboratory. The datasets supporting the conclusions of this article are available in the ProteomeXchange Consortium via the PRIDE partner repository with the dataset identifier PXD051582. The dataset generated during the present study is available upon reasonable request from the corresponding author.

## Abbreviations

CCPCs: child c-kit positive cells
CHD: congenital heart defect
CPCs: c-kit positive cells
CTL: control
ES-CPCs: electrically stimulated c-kit positive cells
GO: gene ontology
HF: heart failure
IPA: ingenuity pathway analysis
MSCs: mesenchymal stromal cells
NCPCs: neonatal c-kit positive cells
PAB: pulmonary artery banding
RV: right ventricular
RVHF: right ventricular heart failure
TAPSE: Tricuspid annular plane systolic excursion
